# Incorporating Medicare Advantage Admissions Into the CMS Hospital-Wide Readmission Measure

**DOI:** 10.1001/jamanetworkopen.2024.14431

**Published:** 2024-06-03

**Authors:** Kelly Kyanko, Kashika M. Sahay, Yongfei Wang, Shu-Xia Li, Michelle Schreiber, Melissa Hager, Raquel Myers, Wanda Johnson, Jing Zhang, Harlan Krumholz, Lisa G. Suter, Elizabeth W. Triche

**Affiliations:** 1 Department of Population Health, New York University Grossman School of Medicine, New York; 2 Yale School of Medicine, New Haven, Connecticut; 3 Yale New Haven Health Services Corporation/Center for Outcomes Research & Evaluation, New Haven, Connecticut; 4 Centers for Medicare & Medicaid Services, Center for Clinical Standards & Quality, Baltimore, Maryland

## Abstract

**Importance:**

Medicare Advantage (MA) enrollment is rapidly expanding, yet Centers for Medicare & Medicaid Services (CMS) claims-based hospital outcome measures, including readmission rates, have historically included only fee-for-service (FFS) beneficiaries.

**Objective:**

To assess the outcomes of incorporating MA data into the CMS claims-based FFS Hospital-Wide All-Cause Unplanned Readmission (HWR) measure.

**Design, Setting, and Participants:**

This cohort study assessed differences in 30-day unadjusted readmission rates and demographic and risk adjustment variables for MA vs FFS admissions. Inpatient FFS and MA administrative claims data were extracted from the Integrated Data Repository for all admissions for Medicare beneficiaries from July 1, 2018, to June 30, 2019. Measure reliability and risk-standardized readmission rates were calculated for the FFS and MA cohort vs the FFS-only cohort, overall and within specialty subgroups (cardiorespiratory, cardiovascular, medicine, surgery, neurology), then changes in hospital performance quintiles were assessed after adding MA admissions.

**Main Outcome and Measure:**

Risk-standardized readmission rates.

**Results:**

The cohort included 11 029 470 admissions (4 077 633 [37.0%] MA; 6 044 060 [54.8%] female; mean [SD] age, 77.7 [8.2] years). Unadjusted readmission rates were slightly higher for MA vs FFS admissions (15.7% vs 15.4%), yet comorbidities were generally lower among MA beneficiaries. Test-retest reliability for the FFS and MA cohort was higher than for the FFS-only cohort (0.78 vs 0.73) and signal-to-noise reliability increased in each specialty subgroup. Mean hospital risk-standardized readmission rates were similar for the FFS and MA cohort and FFS-only cohorts (15.5% vs 15.3%); this trend was consistent across the 5 specialty subgroups. After adding MA admissions to the FFS-only HWR measure, 1489 hospitals (33.1%) had their performance quintile ranking changed. As their proportion of MA admissions increased, more hospitals experienced a change in their performance quintile ranking (147 hospitals [16.3%] in the lowest quintile of percentage MA admissions; 408 [45.3%] in the highest). The combined cohort added 63 hospitals eligible for public reporting and more than 4 million admissions to the measure.

**Conclusions and Relevance:**

In this cohort study, adding MA admissions to the HWR measure was associated with improved measure reliability and precision and enabled the inclusion of more hospitals and beneficiaries. After MA admissions were included, 1 in 3 hospitals had their performance quintile changed, with the greatest shifts among hospitals with a high percentage of MA admissions.

## Key Points

**Question:**

What are the outcomes of incorporating Medicare Advantage (MA) beneficiaries into the Centers for Medicare & Medicaid Services Hospital-Wide Readmission (HWR) measure?

**Findings:**

In this cohort study using data from July 2018 to June 2019 for 11 029 470 Medicare admissions, mean hospital risk-adjusted readmission rates were similar for the combined fee-for-service (FFS) and MA cohort and FFS-only cohort (15.5% vs 15.3%). After adding MA admissions to the FFS-only measure, 1489 (33.1%) of all hospitals and 408 (45.3%) in the highest quintile of MA admissions had a change in performance quintile ranking.

**Meaning:**

These results suggest that adding MA beneficiaries to the HWR measure results in meaningful shifts in hospital performance.

## Teaser

This cohort study investigates the outcomes of incorporating Medicare Advantage data into the Centers for Medicare & Medicaid Services (CMS) claims-based FFS Hospital-Wide All-Cause Unplanned Readmission measure and hospital performance quintile ranking.

## Introduction

The Centers for Medicare & Medicaid Services (CMS) claims-based hospital outcome measures, including hospital readmission rates, have historically only included fee-for-service (FFS) beneficiaries. When these measures were first developed, Medicare Advantage (MA) programs were relatively new and represented a smaller population compared with traditional FFS beneficiaries. Furthermore, MA programs had fewer incentives or checks to support data completeness and timeliness, making their use in quality measurement challenging.^1^ MA enrollment has expanded rapidly in recent years, with half of all Medicare beneficiaries now in MA plans, and growth is projected to continue.^2,3^ Therefore, measures that exclude MA patients may provide an increasingly incomplete assessment of hospital quality, especially for hospitals with a high proportion of MA patients. Recent CMS policies have aimed to improve timeliness, completeness, and accuracy of MA data, thereby enhancing its usability for hospital outcome measures.^4,5^

Moreover, including MA admissions as part of hospital outcome measures addresses stakeholder concerns about differences in readmission outcomes for MA vs FFS beneficiaries potentially attributable to MA plan features.^1,6,7,8,9^ Prior research has suggested that risk profiles of the 2 cohorts may differ, as healthier beneficiaries tend to enroll in MA plans and beneficiaries with higher health care usage may unenroll in MA and switch to FFS Medicare programs.^1,10,11^ However, these trends may be declining with the increasing numbers of enrollees in MA, the revised CMS risk-adjustment model, and changes in policies to prevent favorable selection and plan switching.^1,12^ However, if MA beneficiaries have different readmission rates or risk profiles than FFS beneficiaries and continue to be excluded from CMS hospital outcome measures, using only FFS data will not fully reflect hospital quality across all Medicare beneficiaries. A recent study found that about 1 in 4 top hospitals for readmission and mortality were reclassified to a lower performance group for specific conditions when MA admissions were added, although this study used different methods for measure calculation and risk adjustment than the CMS measures.^7^

To address these issues, we assessed differences in 30-day unadjusted readmission rates and prevalence of demographic and comorbidity risk adjustment variables for MA vs FFS admissions based on CMS’ existing claims-based FFS hospital-wide all-cause unplanned readmission (HWR) measure. We then compared model performance, measure score reliability, risk-standardized readmission rates, and hospital performance for a combined FFS and MA (FFS + MA) cohort vs FFS-only cohort.

## Methods

### Data Sources

This cohort study was conducted under contract to CMS. The Human Investigation Committee at Yale University approved an exemption for this study to use CMS claims and enrollment data and waived the requirement for informed consent because the research involved no more than minimal risk and could not practicably be carried out without the waiver. We followed the Strengthening the Reporting of Observational Studies in Epidemiology (STROBE) reporting guideline.^13^

Only inpatient and enrollment data are used for cohort, outcome, and risk adjustment of the CMS HWR measure. We used a 1-year performance period of July 1, 2018, to June 30, 2019, to align with the CMS reporting year. We extracted 2017-to-2019 inpatient administrative claims data from the Integrated Data Repository (IDR) for all Medicare admissions, including FFS, Medicare Advantage Organization (MAO)-submitted MA inpatient encounters, and hospital-submitted MA claims. MAO-submitted encounter claims are information-only claims (ie, not billing) for items and services provided under the plan that are required to be submitted by MAOs to CMS.^4^ Hospitals that receive disproportionate-share hospital or medical education payments from Medicare are also required to submit information-only claims for inpatient stays for MA beneficiaries, accounting for approximately 90% of all Medicare admissions.^14^

To create the FFS and MA cohort, we combined MAO-submitted and hospital-submitted MA admission claims. We chose this method because MAO-submitted claims capture admissions not found in the hospital-submitted claims, and a small proportion of admissions were only found in the hospital-submitted claims. If an admission was found in both datasets, we used the claim from the hospital-submitted data in our analyses. Hospital-submitted claims are timelier than MAO-submitted claims, which is advantageous for reporting deadlines for CMS measures. Furthermore, unlike MAO-submitted claims, which are associated with a National Provider Identifier (NPI), hospital-submitted claims are already linked with a CMS Certification Number (CCN) used to identify hospitals in CMS measures. For admissions with only MAO-submitted claims, we used IDR Provider History File data to assign the hospital-specific CCN through the NPI. Admissions with only MAO-submitted claims not linked with a CCN were excluded from analyses (<5% of all admissions). Race and ethnicity were not reported due to known inaccuracies in the Medicare administrative data.^15^

### Cohort and Outcomes

The cohort included admissions for beneficiaries enrolled in Medicare FFS or MA aged 65 years or older; discharged alive from a nonfederal short-term acute care or critical access hospital; and not transferred to another acute care or critical access facility. After adding MA beneficiaries, the enrollment requirement was updated to 12 months of FFS or MA enrollment before the index admission and 30 days after index admission; otherwise, cohort inclusion and exclusion criteria remained the same as the FFS-only measure.^16,17^ FFS or MA enrollment was determined based on index admission coverage status. The FFS cohort in these analyses differs slightly from the cohort in the current FFS-only measure. The current FFS-only cohort includes only beneficiaries who were enrolled in FFS for the 12 months before the admission and the 30 days after admission. With the addition of MA data, we were able to include in the FFS cohort admissions with FFS coverage during the admission but who may have switched to MA after admission or had MA coverage at some time during the 12 months prior to admission.

We followed established specifications for the CMS FFS-only HWR measure for risk factors derived from inpatient claims during the 12 months before admission or present at index admission, outcome definitions, and measure score calculations.^16,17^ The HWR measure assesses hospital-level, risk-standardized readmission rate (RSRR) of unplanned, all-cause readmissions within 30 days of hospital discharge, regardless of whether the readmission occurred at the same or different hospitals. A specified set of readmissions are always considered planned and do not count in the outcome (eg, readmissions for bone marrow or organ transplant, maintenance chemotherapy or immunotherapy, rehabilitation, and scheduled procedures). Admissions were excluded if they met any of the following criteria: admitted to a prospective payment system–exempt cancer hospital; without at least 30 days of postdischarge enrollment in Medicare FFS or MA; discharged against medical advice; or admitted for primary psychiatric diagnoses, rehabilitation, or for medical treatment of cancer.

All unplanned readmissions were considered an outcome, regardless of cause. There were several reasons for assessing unplanned readmissions for all causes. From a patient’s perspective, an unplanned readmission for any cause is an adverse event. In addition, making inferences about quality of care based solely on the documented cause of readmission is challenging. Only the first unplanned admission within 30 days of discharge from the index admission is considered a readmission. However, a readmission can be considered an index admission and there can be multiple index admissions per patient in a 30-day period for the HWR measure, providing each meets inclusion criteria. Multiple hospitalizations that result from hospital-to-hospital transfers are considered as a single acute episode of care. Additional information on measure construction available in the eFigure in Supplement 1.

### Statistical Analysis

We first examined risk variable prevalence in MA and FFS admissions, including 1 demographic variable (age) and 31 comorbidities based on condition categories (CCs) for secondary diagnoses and Agency for Healthcare Research and Quality clinical classification software (CCS) condition groups for principal diagnoses.^18^ We then compared observed (unadjusted) readmission rates between MA and FFS admissions overall and in 5 specialty subgroups that reflect how care for patients is organized within hospitals (cardiorespiratory, cardiovascular, medicine, surgery, and neurology). We compared model performance metrics including C statistics and predictive ability in each specialty subgroup and between the 2 cohorts, using patient-level logistic regression models. We also calculated test-retest reliability of hospital measure scores for the total cohort and signal-to-noise reliability for each of the specialty subgroups based on between-hospital variance and hospital volume (see eMethods in Supplement 1 for additional explanation). In general, the higher the hospital volume or between-hospital variance, the higher the signal-to-noise reliability.

For the FFS and MA cohort, we used hierarchical logistic models with a random effect for hospitals to calculate hospital-level standardized readmission ratios (SRRs) for each specialty subgroup, generated the SRR in the overall cohort through the volume-weighted geometric mean of the SRRs of the specialty subgroups, and then produced risk-standardized readmission rates (RSRRs) by multiplying the SRR by the national observed readmission rate. We repeated the aforementioned analyses with FFS-only claims and compared the number of hospitals, number of admissions, and RSRRs from FFS-only and combined FFS and MA data. As with the existing FFS-only HWR measure, results are presented only for hospitals with at least 25 eligible admissions; fewer admissions are not considered reliable for estimating hospital performance and are not publicly reported.^16^

To assess the overall association of adding MA data to hospital measure scores and hospital performance quintile ranking, we examined shifts in hospital RSRR performance quintiles in the FFS-only cohort vs the FFS and MA cohort. To examine associations between hospital characteristics and the addition of MA data, we examined quintile shifts in hospital RSRRs (same quintile, shift of plus or minus 1 quintile, and shift of more than 1 quintile) based on quintiles of the proportion of hospital MA admissions and overall hospital volume. We calculated the Pearson correlation coefficient between the point estimates of hospital RSRRs in the FFS-only cohort and FFS and MA cohort, overall and stratified by quintiles of hospital percentage of MA admissions and quintiles of hospital volume. We then tested the association between additional hospital characteristics obtained from 2018 American Hospital Association Annual Survey data (number of beds, ownership, region, and teaching, critical access, and safety net status) and (1) quintiles of the proportion of hospital MA admissions and (2) hospital performance quintile shifts after the addition of MA data using χ^2^ tests of independence. The threshold for statistical significance was 2-sided *P* < .05. Data analysis was performed using SAS software version 9.4 (SAS Institute) from April 2023 to March 2024.

## Results

Among the 11 029 470 admissions in the study cohort, which included all admissions for Medicare beneficiaries from July 1, 2018, to June 30, 2019, 4 077 633 (37.0%) were MA, 6 044 060 (54.8%) were female, and mean (SD) age was 77.7 (8.2) years. Mean (SD) age was 77.3 (7.9) years for MA admissions vs 77.9 (8.3) years for FFS admissions (Table 1). The prevalences of comorbidities were similar but generally lower among MA admissions than FFS, with a mean difference between FFS and MA comorbidities of 0.7 percentage points. Disorders of fluid, electrolyte, and acid-base were more common in FFS admissions (22.0% FFS vs 19.3% MA; difference of 2.8 percentage points), and diabetes was more prevalent among MA admissions (37.0% FFS vs 40.1% MA; difference of −3.1 percentage points). Observed (unadjusted) 30-day readmission rates were lower for FFS admissions than MA admissions for the overall cohort (15.4% vs 15.7%; difference of −0.4 percentage points) (Table 2).

**Table 1.** Demographic and Comorbidity Risk Model Variables Among Admissions for Fee-for-Service vs Medicare Advantage Admissions

Description	Admissions, No. (%)		Difference, FFS − MA, percentage points
	FFS (n = 6 951 837)	MA (n = 4 077 633)	
Age, mean (SD), y	77.9 (8.3)	77.3 (7.9)	0.6
Comorbidities^a^			
Metastatic cancer/acute leukemia (CC 8)	250 667 (3.6)	136 660 (3.4)	0.3
Severe cancer (CC 9, 10)	380 837 (5.5)	206 319 (5.1)	0.4
Other cancers (CC 11-14)	465 493 (6.7)	260 895 (6.4)	0.3
Severe hematological disorders (CC 46)	59 530 (0.9)	29 312 (0.7)	0.1
Coagulation defects and other specified hematological disorders (CC 48)	279 993 (4.0)	147 051 (3.6)	0.4
Iron deficiency or other unspecified anemia and blood disease (CC 49)	2 858 573 (41.1)	1 582 949 (38.8)	2.3
End-stage liver disease (CC 27, 28)	187 868 (2.7)	112 428 (2.8)	−0.1
Pancreatic disease (CC 34, 36)	416 772 (6.0)	216 518 (5.3)	0.7
Dialysis status (CC 134)	135 020 (1.9)	54 422 (1.3)	0.6
Acute kidney failure (CC 135-140)	2 441 097 (35.1)	1 425 848 (35.0)	0.2
Transplants (CC 132, 186)	62 120 (0.9)	22 155 (0.5)	0.4
Severe infection (CC 1, 3-6)	78 726 (1.1)	43 832 (1.1)	0.1
Other infectious disease and pneumonias (CC 7, 114-116)	1 312 530 (18.9)	676 909 (16.6)	2.3
Septicemia/shock (CC 2)	478 123 (6.9)	244 358 (6.0)	0.9
Congestive heart failure (CC 85)	1 279 305 (18.4)	688 258 (16.9)	1.5
Coronary atherosclerosis or angina, cerebrovascular disease (CC 86-89, 102, 105-109)	3 208 668 (46.2)	1 802 070 (44.2)	2.0
Specified arrhythmias (CC 96, 97)	1 249 378 (18.0)	627 875 (15.4)	2.6
Cardiorespiratory failure or cardiorespiratory shock (CC 84)	760 776 (10.9)	404 634 (9.9)	1.0
Coronary obstructive pulmonary disease (CC 111)	1 808 252 (26.0)	1 050 950 (25.8)	0.2
Fibrosis of lung or other chronic lung disorders (CC 112)	205 712 (3.0)	111 656 (2.7)	0.2
Protein-calorie malnutrition (CC 21)	765 232 (11.0)	417 123 (10.2)	0.8
Disorders of fluid, electrolyte, acid-base (CC 23, 24)	1 531 359 (22.0)	785 254 (19.3)	2.8
Rheumatoid arthritis and inflammatory connective tissue disease (CC 40)	388 156 (5.6)	199 193 (4.9)	0.7
Diabetes (CC 17-19, 122, 123)	2 575 065 (37.0)	1 635 742 (40.1)	−3.1
Ulcers (CC 157-161)	345 438 (5.0)	184 435 (4.5)	0.5
Hemiplegia, paraplegia, paralysis, functional disability (CC 70, 71, 73, 74, 103, 104, 189, 190)	330 096 (4.8)	168 402 (4.1)	0.6
Seizure disorders and convulsions (CC 79)	289 387 (4.2)	150 421 (3.7)	0.5
Respirator dependence/tracheostomy status (CC 82)	20 103 (0.3)	9784 (0.2)	0.1
Drug and alcohol disorders (CC 54, 55)	205 111 (3.0)	124 296 (3.1)	−0.1
Psychiatric comorbidity (CC 57-59, 61, 63)	1 925 953 (27.7)	1 031 480 (25.3)	2.4
Hip fracture/dislocation (CC 170)	111 520 (1.6)	52 733 (1.3)	0.3

Abbreviations: CC, condition category; FFS, fee-for-service; MA, Medicare Advantage.

^a^ CC grouping of *International Statistical Classification of Diseases, Tenth Revision, Clinical Modification (ICD-10-CM)* diagnosis codes in clinically relevant categories.

**Table 2.** Admissions and Observed (Unadjusted) 30-Day Readmission Rates for Medicare Advantage vs Fee-for-Service Admissions, Overall and by 5 Specialty Subgroups

Specialty subgroups	Total		FFS		MA		Difference, FFS − MA, percentage points
	Total admissions, No.	Readmissions, No. (%)	Total admissions, No.	Readmissions, No. (%)	Total admissions, No.	Readmissions, No. (%)	
Overall	11 029 470	1 707 854 (15.5)	6 951 837	1 066 775 (15.4)	4 077 633	641 079 (15.7)	−0.4
Cardiorespiratory	1 306 739	240 690 (18.4)	830 142	151 693 (18.3)	476 597	88 997 (18.7)	−0.4
Cardiovascular	1 158 878	168 331 (14.5)	718 090	102 637 (14.3)	440 788	65 694 (14.9)	−0.6
Medicine	5 306 659	941 265 (17.7)	3 356 886	589 831 (17.6)	1 949 773	351 434 (18.0)	−0.5
Neurology	645 101	83 493 (12.9)	397 740	50 235 (12.6)	247 361	33 258 (13.5)	−0.8
Surgical	2 612 093	274 075 (10.5)	1 648 979	172 379 (10.5)	963 114	101 696 (10.6)	−0.1

Abbreviations: FFS, fee-for-service; MA, Medicare Advantage.

Details of model performance for the combined FFS and MA cohort vs the FFS-only cohort are in eTable 1 in Supplement 1. The C statistics for the FFS and MA cohort ranged from 0.60 to 0.69 for each of the 5 specialty subgroups and were similar to the FFS-only cohort (≤0.02 difference in C statistics between cohorts). Predictive ability was also similar between the combined FFS and MA and FFS-only cohorts. eTable 2 in Supplement 2 shows the hierarchical logistic regression model parameter estimates for the 5 specialty subgroups in the FFS and MA cohort.

When we added MA data, 63 additional hospitals met the threshold for public reporting (at least 25 admissions). For each specialty subgroup, more hospitals were included in the FFS and MA data compared with the FFS-only data (eTable 3 in Supplement 1). Test-retest reliability for the overall cohort was 0.780 in the combined FFS and MA cohort vs 0.725 in the FFS-only cohort. Median signal-to-noise ratio (calculated based on between-hospital variance and hospital volume for each specialty subgroup) was higher in the FFS and MA cohort (range across subgroups, 0.63-0.82) than the FFS-only cohort (range across subgroups, 0.54-0.74). Between-hospital variance ranged from 0.024 to 0.034 across specialty subgroups for the FFS and MA cohort and was similar in the FFS-only cohort.

Table 3 presents distribution of hospital index admission volume and RSRR for hospitals with 25 or more eligible admissions within each specialty subgroup. For each specialty subgroup, admission volume was higher in the FFS and MA cohort than in the FFS-only cohort. Overall risk-standardized readmission rates were similar for the FFS and MA cohort and FFS-only cohort (mean 15.5% vs 15.3%). This trend was seen across all specialty subgroups.

**Table 3.** Hospital Volume and RSRRs for FFS and MA Cohort and FFS-Only Cohort, Overall and by Specialty Subgroup

Specialty subgroup	FFS and MA cohort		FFS-only cohort	
	Mean (SD)	Median (IQR)	Mean (SD)	Median (IQR)
Overall^a^				
Hospital volume	2417 (3269)	995 (253-3546)	1544 (2073)	650 (200-2172)
RSRR (%)	15.5 (1.3)	15.4 (14.7-16.1)	15.3 (1.2)	15.3 (14.7-15.9)
Cardiorespiratory				
Hospital volume	322 (333)	203 (83-460)	212 (210)	135 (64-294)
RSRR (%)	18.5 (1.8)	18.3 (17.3-19.5)	18.3 (1.6)	18.2 (17.3-19.2)
Cardiovascular				
Hospital volume	401 (430)	267 (89-562)	270 (274)	183 (71-365)
RSRR (%)	14.6 (1.3)	14.5 (13.8-15.3)	14.3 (1.0)	14.3 (13.7-14.9)
Medicine				
Hospital volume	1225 (1561)	592 (149-1840)	790 (985)	380 (122-1129)
RSRR (%)	17.8 (1.9)	17.6 (16.7-18.8)	17.6 (1.7)	17.5 (16.6-18.5)
Neurology				
Hospital volume	248 (248)	167 (72-337)	168 (156)	116 (59-226)
RSRR (%)	13.0 (1.3)	12.9 (12.2-13.7)	12.7 (1.2)	12.6 (11.9-13.3)
Surgical				
Hospital volume	793 (984)	440 (148-1074)	519 (657)	277 (104-677)
RSRR (%)	10.5 (1.0)	10.4 (9.9-11.1)	10.5 (0.9)	10.4 (10.0-11.0)

Abbreviations: FFS, fee-for-service; MA, Medicare Advantage; RSRR, risk-standardized readmission rates.

^a^ Includes all specialty subgroups, for hospitals with 25 or more admissions in each subgroup.

Table 4 shows hospital performance quintile shifts in RSRR in the FFS and MA cohort compared with the FFS-only cohort. After adding MA admissions to the FFS-only HWR measure, 1489 hospitals (33.1%) changed their performance quintile ranking; 1283 (28.5%) moved within plus or minus 1 performance quintile, and 206 (4.6%) moved greater than 1 quintile. Correlation between hospital RSRRs was 0.92. As hospitals’ proportion of MA admissions increased, more hospitals changed their performance quintile ranking (147 hospitals [16.3%] in the lowest quintile of percentage MA admissions; 408 [45.3%] in the highest quintile of percentage of MA admissions). As hospital volume increased, trends in RSRR shifts were less pronounced (229 hospitals [25.5%] in lowest volume quintile changed performance quintile ranking; 307 hospitals [34.1%] in the highest volume quintile). Nongovernment-owned not-for-profit and for-profit hospitals, hospitals in the northeastern US, teaching hospitals, non–critical access hospitals (ie, nonrural), and non–safety net hospitals had larger performance shifts (>1 quintile) after the addition of MA admissions to their measure scores (Table 5).^19^ This could be partially explained by the higher proportion of MA admissions (eTable 4 in Supplement 1) among hospitals with these characteristics. Hospital bed size was not significantly associated with shifts in performance quintiles.

**Table 4.** Shifts in RSRR Hospital Performance Quintile Rankings, Overall and Based on Hospitals’ Percentages of MA Admissions and Total Admission Volume^a^

Description	No. of hospitals	No. (%)			Correlation^b^
		Same	±1	>1	
Overall^c^	4500	3011 (66.9)	1283 (28.5)	206 (4.6)	0.92
By % of MA admissions					
Q1: 0.00%-12.51%	900	753 (83.7)	146 (16.2)	1 (0.1)	0.99
Q2: 12.51%-23.37%	900	647 (71.9)	242 (26.9)	11 (1.2)	0.96
Q3: 23.39%-32.39%	900	586 (65.1)	281 (31.2)	33 (3.7)	0.93
Q4: 32.41%-44.01%	900	533 (59.2)	315 (35.0)	52 (5.8)	0.91
Q5: 44.03%-95.88%	900	492 (54.7)	299 (33.2)	109 (12.1)	0.81
By FFS and MA cohort admission volume					
Q1: 25-198 admissions	899	670 (74.5)	220 (24.5)	9 (1.0)	0.93
Q2: 199-583 admissions	900	603 (67.0)	259 (28.8)	38 (4.2)	0.89
Q3: 584-1842 admissions	901	563 (62.5)	290 (32.2)	48 (5.3)	0.94
Q4: 1844-4321 admissions	900	582 (64.7)	265 (29.4)	53 (5.9)	0.90
Q5: 4325-40 801 admissions	900	593 (65.9)	249 (27.7)	58 (6.4)	0.90

Abbreviations: FFS, fee-for-service; MA, Medicare Advantage; Q, quintile; RSRR, risk-standardized readmission rate.

^a^ Columns represent the number and percentage of hospitals that stayed in their same ranking, within 1 ranking, or moved greater than 1 performance quintile ranking after the addition of MA admissions.

^b^ Correlation refers to the Pearson correlation coefficient between the point estimates of hospital RSRRs in the FFS-only and FFS and MA cohorts.

^c^ Includes hospitals with 25 or more FFS admissions.

**Table 5.** Shifts in Risk-Standardized Readmission Rate Hospital Performance Quintile Rankings by Hospital Characteristics^a^

Characteristic	Total No. of hospitals	Quintile, No. (%)			*P* value^b^
		Same	±1	>1	
Overall	4390	2948 (67.2)	1242 (28.3)	200 (4.6)	NA
No. of beds					
<300	3649	2453 (67.2)	1032 (28.3)	164 (4.5)	.24
300 to 600	560	377 (67.3)	151 (27.0)	32 (5.7)	
>600	181	118 (65.2)	59 (32.6)	4 (2.2)	
Ownership					
Government	940	694 (73.8)	221 (23.5)	25 (2.7)	<.001
Not-for-profit	2658	1737 (65.3)	786 (29.6)	135 (5.1)	
For profit	792	517 (65.3)	235 (29.7)	40 (5.1)	
Region^c^					
Northeast	505	321 (63.6)	149 (29.5)	35 (6.9)	.01
Midwest	1332	879 (66.0)	395 (29.7)	58 (4.4)	
South	1638	1127 (68.8)	449 (27.4)	62 (3.8)	
West	864	595 (68.9)	228 (26.4)	41 (4.7)	
Teaching status^d^					
COTH	225	144 (64.0)	72 (32.0)	9 (4.0)	.008
Non-COTH teaching	1331	879 (66.0)	369 (27.7)	83 (6.2)	
Nonteaching	2834	1925 (67.9)	801 (28.3)	108 (3.8)	
Critical access hospital^e^					
No	3141	2031 (64.7)	933 (29.7)	177 (5.6)	<.001
Yes	1249	917 (73.4)	309 (24.7)	23 (1.8)	
Safety net hospital^c^^,^^f^					
No	3130	2039 (65.1)	933 (29.8)	158 (5.0)	<.001
Yes	1259	908 (72.1)	309 (24.5)	42 (3.3)	

Abbreviation: COTH, Association of American Medical Colleges Council of Teaching Hospitals and Health Systems.

^a^ Columns represent the number and percentage of hospitals that stayed in their same ranking, within 1 ranking, or moved greater than 1 performance quintile ranking after the addition of MA admissions. Hospital characteristics were obtained from 2018 American Hospital Association Annual Survey data. Analyses excluded 110 hospitals that did not link to AHA data.

^b^ Significance was measured by χ^2^ tests of independence.

^c^ Numbers and percentages do not sum to total for region (missing n = 51) and safety net hospital (missing n = 1). Missing data were included as a category for χ^2^ tests for these variables.

^d^ COTH hospitals are major teaching hospitals with membership in the Association of American Medical Colleges Council of Teaching Hospitals and Health Systems (COTH).

^e^ Critical Access Hospitals are rural hospitals that maintain no more than 25 beds and are located more than 35 miles from the nearest hospital, among other criteria.^23^

^f^ Safety net hospital defined as government hospital or nongovernment hospital with high Medicaid caseload.

## Discussion

With the inclusion of Medicare Advantage admissions, 63 additional hospitals and more than 4 million additional admissions would be eligible for public reporting for the CMS claims-based HWR measure. Reliability increased overall and among each of the 5 specialty subgroups. Mean risk-standardized readmission rates were similar for the FFS and  MA cohort and FFS-only cohort; however, 1 in 3 hospitals changed their performance quintile ranking after the addition of MA admissions. Hospitals with a high proportion of MA admissions were more likely to experience significant performance shifts after the addition of MA admissions to their measure scores; for hospitals in the highest quintile of percentage of MA admissions, just under half (45.3%) had their performance quintile change. Hospital characteristics also found in those with a high proportion of MA admissions (nongovernment-owned not-for-profit and for-profit, northeastern US, teaching, nonrural, and non–safety net hospitals) were associated with performance shifts.

Unadjusted readmissions were slightly higher for MA than FFS admissions, and hospital risk–adjusted readmission rates were similar for the FFS and MA and FFS-only cohorts. These findings are in contrast to previous studies that reported mixed results or generally lower adjusted readmission rates for MA beneficiaries.^6,7,8,20,21^ A recent study using 2018 data found 0.4% to 0.7% lower adjusted readmission rates across 4 conditions for a FFS and MA cohort compared with a FFS-only cohort.^7^ This difference in findings could be attributable to changes in the risk profiles and outcomes of MA beneficiaries over time,^21^ differences in how outcomes and risk adjustment were calculated, previous limitations to a single MAO or geographic area, or differences in data sources.

Strengths of our study include the use of established and validated measure specifications used in CMS reporting and payment programs. By combining hospital- and MAO-submitted claims, our dataset encompasses all Medicare admissions, rather than relying on incomplete MedPAR data used by some previous studies,^8,9,21^ which only includes claims for MA admissions submitted by hospitals that receive disproportionate-share hospital or medical education payments from Medicare. By preferentially using hospital-submitted claims over MAO-submitted claims for admissions in both data sources, we leverage the improved timeliness of the hospital-submitted claims.

Prior research has found evidence of more intensive use of certain diagnosis codes (ie, upcoding) for MA beneficiaries leading to higher risk scores used for payment based on the hierarchical condition categories (HCCs).^1,22^ In contrast, the risk variables we use in the HWR measure are from inpatient claims only, are not used for payment, and do not apply the hierarchical methodology for the condition categories; rather, they are based on clinically relevant condition categories for secondary diagnoses and Agency for Healthcare Research and Quality clinical classification software (CCS) condition groups for principal diagnoses. Using this approach, the prevalence of comorbidities was slightly lower among MA admissions than FFS. This suggests concerns about upcoding should not influence the decision to include MA beneficiaries in hospital outcome measures that rely on inpatient data only. However, given the evidence and the application of HWR across CMS measurement programs, incorporating MA data into the HWR measure may require continued monitoring.

CMS plans to use the expanded FFS and MA cohort for public reporting of 30-day readmission rates for US hospitals in an updated version of the readmission measure that incorporates both electronic health record (EHR) data and claims.^23^ This hybrid HWR measure is identical to the claims-based HWR measure but adds 10 clinical data elements from the EHR for risk adjustment to improve model performance.^24^ Measure scores based on the claims-only model were highly correlated with those based on the hybrid model (correlation coefficient = 0.99). CMS proposed to modify the cohort to include MA beneficiaries in the fiscal year 2024 Inpatient Prospective Payment System Proposed Rule for the fiscal year 2027 payment determination and subsequent years.

### Limitations

This study has limitations. Our method for combining both MAO- and hospital-submitted claims is novel and would benefit from tests for external validation and reproducibility. As policies continue to be implemented to improve the usability and timeliness of MAO-submitted encounter data, the need for this approach should be reexamined. Furthermore, a small number of admissions (<5% in 2018 data) found only in MAO-submitted data were not associated with an NPI and were therefore excluded from analyses. Our analyses were based on pre–COVID-19 data for initial assessment of the outcomes of including MA admissions; minor changes in coding practices, hospital performance, and/or clinical care during COVID-19 might affect our results.

## Conclusions

This cohort study found that incorporating MA admissions into the current HWR measure was associated with improved reliability, captured data from more hospitals and beneficiaries, and improved visibility of quality of care for the substantial and growing population of patients in MA plans. Adding MA beneficiaries to the CMS HWR measure resulted in shifts in hospital performance rankings, especially among hospitals with a high proportion of MA admissions.

## Notes

Supplement 1. **eMethods. **Additional Detail on Reliability **eFigure. **HWR Flow Diagram of Inclusion and Exclusion Criteria and Specialty Cohort Assignment for the Index Admission **eTable 1. **Predictive Ability and C-Statistics for Admission-Level Models for the FFS+MA and the FFS-Only Cohorts by Specialty Subgroup **eTable 3. **Signal-to-Noise Reliability and Between-Hospital Variance in the FFS+MA and FFS-Only Cohorts **eTable 4. **Quintiles of Percent Medicare Advantage Admissions by Hospital Characteristics
eReferences

Supplement 2. **eTable 2. **Hierarchical Logistic Regression Model Parameter Estimates for Five HWR Specialty Subgroups: FFS+MA Cohort

Supplement 3.
Data Sharing Statement
