# Supplementary material for: Incorporating Medicare Advantage Admissions Into the CMS Hospital-Wide Readmission Measure
Source: JAMA Netw Open. 2024 Jun 3;7(6):e2414431. doi: 10.1001/jamanetworkopen.2024.14431 (PMC11148674; doi:10.1001/jamanetworkopen.2024.14431)
Supplement: Supplement 1. — eMethods. Additional Detail on Reliability eFigure. HWR Flow Diagram of Inclusion and Exclusion Criteria and Specialty Cohort Assignment for the Index Admission eTable 1. Predictive Ability and C-Statistics for Admission-Level Models for the FFS+MA and the FFS-Only Cohorts by Specialty Subgroup eTable 3. Signal-to-Noise Reliability and Between-Hospital Variance in the FFS+MA and FFS-Only Cohorts eTable 4. Quintiles of Percent Medicare Advantage Admissions by Hospital Characteristics eReferences [file jamanetwopen-e2414431-s001.pdf]

Supplemental Online Content

Kyanko K, Sahay KM, Wang Y, et al. Incorporating Medicare Advantage admissions into the CMS Hospital-Wide Readmission measure. *JAMA Netw Open.* 2024;7(6):e2414431. doi:10.1001/jamanetworkopen.2024.14431

eMethods. Additional Detail on Reliability

eFigure. HWR Flow Diagram of Inclusion and Exclusion Criteria and Specialty Cohort Assignment for the Index Admission

eTable 1. Predictive Ability and C-Statistics for Admission-Level Models for the FFS+MA and the FFS-Only Cohorts by Specialty Subgroup

eTable 3. Signal-to-Noise Reliability and Between-Hospital Variance in the FFS+MA and FFS-Only Cohorts

eTable 4. Quintiles of Percent Medicare Advantage Admissions by Hospital Characteristics

eReferences

This supplemental material has been provided by the authors to give readers additional information about their work.

© 2024 Kyanko K et al. *JAMA Network Open.*

eMethods: Additional Detail on Reliability

Measure reliability is a way to see if the measure is consistently producing similar results when it is repeated more than once. The results tell us whether the measure results are consistent over time and not random. We performed two types of reliability testing. First, we estimated the overall measure score reliability by calculating the intra-class correlation coefficient (ICC) using a split sample (i.e. test-retest) method. Second, we estimated the facility-level reliability (signal-to-noise reliability) separately for each of the 5 specialty subgroups.

For test-retest reliability, we randomly sampled half of patients within each cohort and each hospital, calculated the measure score for each hospital using the first half of sample, and then repeated the calculation using the second half. The extent that the calculated measure scores of these two subsets agree provide evidence that the measure is assessing an attribute of the hospital, not of the patients. As a metric of agreement, we calculated the intra-class correlation coefficient,[1] and assessed the values according to conventional standards.[2] For test-retest reliability, as cohort size increases, the two random half samples are more likely to be similar to each other. This will result in a higher agreement (reliability) score.

Signal to noise ratio (STN) is another metric of reliability. STN is a ratio of meaningful information (signal variance) compared to random variation (noise variance). A higher signal to noise ratio shows the information we are meaning to measure is stronger than the random variation. To calculate the STN, we estimated the signal variance using hierarchical logistic regression. Since we fit the hierarchical logistic regression models separately for each of the 5 specialty subgroups, STN is only estimated for each subgroup rather than for the overall measure. STN reliability is calculated as between hospital variance divided by the sum of between hospital variance and noise variance. Noise variance decreases as volume increases, so the STN reliability increases as the cohort size increases even when the between-hospital variances (signal variances) stay the same. Usually, an increase in cohort size will result in a higher agreement (reliability) score.

© 2024 Kyanko K et al. *JAMA Network Open.*

eFigure: HWR Flow Diagram of Inclusion and Exclusion Criteria and Specialty Cohort Assignment for the Index Admission[a]

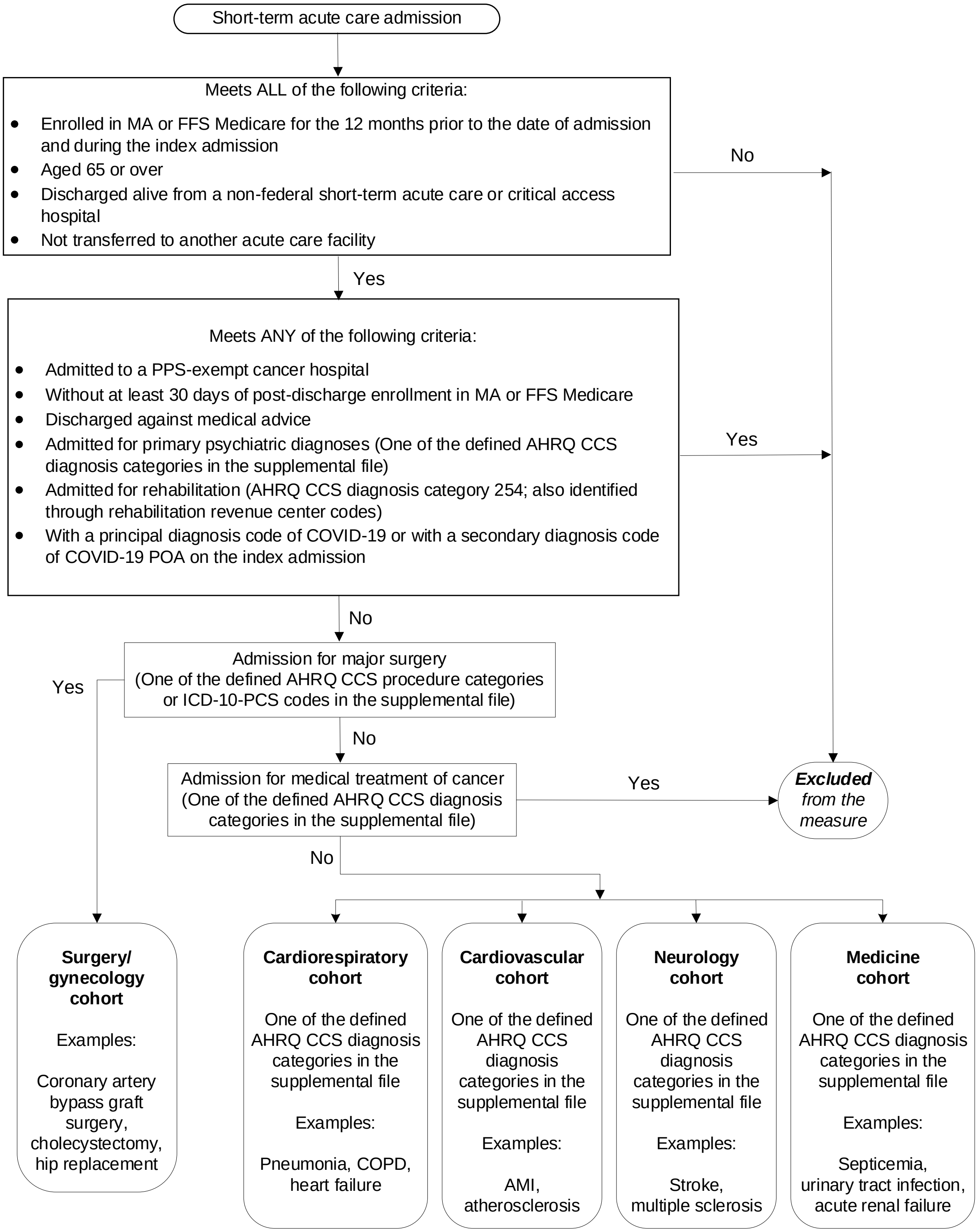

[a] Figure adapted from: Debuhr J, Grady JN, Norton E, et al. 2023 Hospital-Wide Readmission Measure Updates and Specifications Report, Version 12.0. https://qualitynet.cms.gov/files/645064349920e9001651f24d?filename=2023_HWR_AUS_Report_v1.0.pdf

© 2024 Kyanko K et al. *JAMA Network Open.*

eTable 1: Predictive Ability and C-Statistics for Admission-Level Models for the FFS+MA and the FFS-Only Cohorts by Specialty Subgroup

Specialty Subgroup	FFS+MA Cohort		FFS-Only Cohort	
	Predictive Ability % (lowest decile – highest decile)	c-statistic	Predictive Ability % (lowest decile – highest decile)	c-statistic
**Cardiorespiratory**	10.6-28.6	0.60	10.3-28.6	0.60
**Cardiovascular**	8.0-25.4	0.62	7.3-25.6	0.63
**Medicine**	9.6-29.3	0.61	9.2-29.3	0.62
**Neurology**	7.8-22.3	0.60	6.7-22.5	0.62
**Surgical**	2.3-23.9	0.69	2.2-24.0	0.70

FFS=fee-for-service; MA=Medicare Advantage

© 2024 Kyanko K et al. *JAMA Network Open.*

eTable 3: Signal-to-Noise Reliability and Between-Hospital Variance in the FFS+MA and FFS-Only Cohorts

Specialty Cohort	Combined FFS+MA Cohort			FFS-Only Cohort		
	N1[a]	Median Signal-to-Noise Ratio (IQR)	Between-Hospital Variance	N2[b]	Median Signal-to-Noise Ratio (IQR)	Between-Hospital Variance
**Cardiorespiratory**	4,043	0.64 (0.42-0.80)	0.029	3,886	0.54 (0.36-0.72)	0.029
**Cardiovascular**	2,858	0.67 (0.40-0.81)	0.025	2,602	0.54 (0.32-0.70)	0.021
**Medicine**	4,328	0.82 (0.54-0.94)	0.026	4,242	0.74 (0.47-0.89)	0.024
**Neurology**	2,539	0.63 (0.42-0.78)	0.034	2,265	0.55 (0.38-0.70)	0.035
**Surgical**	3,286	0.76 (0.52-0.89)	0.024	3,165	0.66 (0.42-0.83)	0.023

[a] N1: Number of hospitals with at least 25 FFS+MA admissions (4,563); [b] N2: number of hospitals with at least 25 FFS admissions (4,500).
Test-retest reliability for the overall cohort was 0.780 in the FFS+MA cohort versus 0.725 in the FFS-only cohort.
FFS=fee-for-service; IQR = interquartile range; MA=Medicare Advantage.

© 2024 Kyanko K et al. *JAMA Network Open.*

eTable 4: Quintiles of Percent Medicare Advantage Admissions by Hospital Characteristics[a, b]

Description	Total<br><br>No.<br>(N=4,390)	First Quintile<br>No. (%)<br>(N=885)	Second Quintile<br>No. (%)<br>(N=878)	Third Quintile<br>No. (%)<br>(N=889)	Fourth Quintile<br>No. (%)<br>(N=872)	Fifth Quintile<br>No. (%)<br>(N=866)
Number of beds						
< 300	3,649	868 (23.8)	780 (21.4)	739 (20.3)	649 (17.8)	613 (16.8)
300 to 600	560	16 (2.9)	71 (12.7)	115 (20.5)	160 (28.6)	198 (35.4)
> 600	181	1 (0.6)	27 (14.9)	35 (19.3)	63 (34.8)	55 (30.4)
Ownership						
Government	940	368 (39.1)	205 (21.8)	174 (18.5)	106 (11.3)	87 (9.3)
Not-for-profit	2,658	428 (16.1)	524 (19.7)	552 (20.8)	574 (21.6)	580 (21.8)
For profit	792	89 (11.2)	149 (18.8)	163 (20.6)	192 (24.2)	199 (25.1)
Region[c]						
Northeast	505	23 (4.6)	90 (17.8)	124 (24.6)	119 (23.6)	149 (29.5)
Midwest	1,332	96 (7.2)	271 (20.3)	294 (22.1)	388 (29.1)	283 (21.2)
South	1,638	493 (30.1)	371 (22.6)	364 (22.2)	237 (14.5)	173 (10.6)
West	864	267 (30.9)	146 (16.9)	107 (12.4)	128 (14.8)	216 (25.0)
Teaching status[d]						
COTH	225	4 (1.8)	37 (16.4)	40 (17.8)	74 (32.9)	70 (31.1)
Non-COTH teaching	1,331	109 (8.2)	193 (14.6)	297 (22.3)	331 (24.9)	401 (30.1)
Non-teaching	2,834	772 (27.2)	648 (22.9)	552 (19.5)	467 (16.5)	395 (13.9)
Critical Access Hospital[e]						
No	3,141	311 (9.9)	566 (18.0)	689 (21.9)	766 (24.4)	809 (25.8)
Yes	1,249	574 (46.0)	312 (25.0)	200 (16.0)	106 (8.5)	57 (4.6)
Safety Net Hospital[c, f]						
No	3,130	462 (14.8)	614 (19.6)	654 (20.9)	704 (22.5)	696 (22.2)
Yes	1,259	422 (33.5)	264 (21.0)	235 (18.7)	168 (13.3)	170 (13.5)

[a] Columns represent quintiles of percent MA of total FFS+MA admissions. Hospital characteristics were obtained from 2018 American Hospital Association (AHA) Annual Survey data. Analyses excluded 110 hospitals that did not link to AHA data.

[b] Significance was measured by chi-square tests of independence. P-values for all characteristics <0.001.

[c] N's and percents do not sum to total for Region (missing N=51) and Safety net hospital (missing N=1). Missing data were included as a category for chi-square tests for these variables.

[d] COTH hospitals are major teaching hospitals with membership in the Association of American Medical Colleges Council of Teaching Hospitals and Health Systems (COTH).

[e] Critical Access Hospitals are rural hospitals that maintain no more than 25 beds and are located more than 35-miles from the nearest hospital, among other criteria[3]

[f] Safety net hospital defined as government hospital or non-government hospital with high Medicaid caseload.

© 2024 Kyanko K et al. *JAMA Network Open.*

eReferences

1. Shrout PE, Fleiss JL. Intraclass correlations: uses in assessing rater reliability. *Psychological bulletin*. 1979;86(2):420.
2. Landis JR, Koch GG. The measurement of observer agreement for categorical data. *biometrics*. 1977:159-174.
3. Centers for Medicare & Medicaid Services (CMS). Certification & compliance: Critical Access Hospitals. Accessed March 18, 2024. https://www.cms.gov/medicare/health-safety-standards/certification-compliance/critical-access-hospitals

© 2024 Kyanko K et al. *JAMA Network Open.*
