# Supplementary material for: Incorporating Medicare Advantage Admissions Into the CMS Hospital-Wide Readmission Measure
Source: JAMA Netw Open. 2024 Jun 3;7(6):e2414431. doi: 10.1001/jamanetworkopen.2024.14431 (PMC11148674; doi:10.1001/jamanetworkopen.2024.14431)
Supplement: Supplement 3. — Data Sharing Statement [file jamanetwopen-e2414431-s003.pdf]

Data Sharing Statement

Kyanko. Incorporating Medicare Advantage Admissions Into the CMS Hospital-Wide Readmission Measure. *JAMA Netw Open*. Published June 03, 2024. doi:10.1001/jamanetworkopen.2024.14431

Data
Data available: No
